# Genome and clonal hematopoiesis stability contrasts with immune, cfDNA, mitochondrial, and telomere length changes during short duration spaceflight

**DOI:** 10.1093/pcmedi/pbae007

**Published:** 2024-04-08

**Authors:** J Sebastian Garcia-Medina, Karolina Sienkiewicz, S Anand Narayanan, Eliah G Overbey, Kirill Grigorev, Krista A Ryon, Marissa Burke, Jacqueline Proszynski, Braden Tierney, Caleb M Schmidt, Nuria Mencia-Trinchant, Remi Klotz, Veronica Ortiz, Jonathan Foox, Christopher Chin, Deena Najjar, Irina Matei, Irenaeus Chan, Carlos Cruchaga, Ashley Kleinman, JangKeun Kim, Alexander Lucaci, Conor Loy, Omary Mzava, Iwijn De Vlaminck, Anvita Singaraju, Lynn E Taylor, Julian C Schmidt, Michael A Schmidt, Kelly Blease, Juan Moreno, Andrew Boddicker, Junhua Zhao, Bryan Lajoie, Andrew Altomare, Semyon Kruglyak, Shawn Levy, Min Yu, Duane C Hassane, Susan M Bailey, Kelly Bolton, Jaime Mateus, Christopher E Mason

**Affiliations:** Department of Physiology and Biophysics, Weill Cornell Medicine, Cornell University, New York, NY 10021, USA; The HRH Prince Alwaleed Bin Talal Bin Abdulaziz Alsaud Institute for Computational Biomedicine, Weill Cornell Medicine, New York, NY 10021, USA; Department of Physiology and Biophysics, Weill Cornell Medicine, Cornell University, New York, NY 10021, USA; The HRH Prince Alwaleed Bin Talal Bin Abdulaziz Alsaud Institute for Computational Biomedicine, Weill Cornell Medicine, New York, NY 10021, USA; Department of Nutrition and Integrative Physiology, Florida State University, Tallahassee, FL 32306, USA; Department of Physiology and Biophysics, Weill Cornell Medicine, Cornell University, New York, NY 10021, USA; The HRH Prince Alwaleed Bin Talal Bin Abdulaziz Alsaud Institute for Computational Biomedicine, Weill Cornell Medicine, New York, NY 10021, USA; BioAstra Inc, New York, NY, USA; Department of Physiology and Biophysics, Weill Cornell Medicine, Cornell University, New York, NY 10021, USA; The HRH Prince Alwaleed Bin Talal Bin Abdulaziz Alsaud Institute for Computational Biomedicine, Weill Cornell Medicine, New York, NY 10021, USA; Department of Physiology and Biophysics, Weill Cornell Medicine, Cornell University, New York, NY 10021, USA; Department of Physiology and Biophysics, Weill Cornell Medicine, Cornell University, New York, NY 10021, USA; Department of Physiology and Biophysics, Weill Cornell Medicine, Cornell University, New York, NY 10021, USA; Department of Physiology and Biophysics, Weill Cornell Medicine, Cornell University, New York, NY 10021, USA; The HRH Prince Alwaleed Bin Talal Bin Abdulaziz Alsaud Institute for Computational Biomedicine, Weill Cornell Medicine, New York, NY 10021, USA; Sovaris Aerospace, Boulder, CO 80302, USA; Advanced Pattern Analysis & Human Performance Group, Boulder, CO 80302, USA; Department of Systems Engineering, Colorado State University, Fort Collins, CO 80523, USA; Department of Physiology and Biophysics, Weill Cornell Medicine, Cornell University, New York, NY 10021, USA; Department of Stem Cell Biology and Regenerative Medicine, Keck School of Medicine, University of Southern California, Los Angeles, CA 90033, USA; Department of Stem Cell Biology and Regenerative Medicine, Keck School of Medicine, University of Southern California, Los Angeles, CA 90033, USA; Department of Physiology and Biophysics, Weill Cornell Medicine, Cornell University, New York, NY 10021, USA; The HRH Prince Alwaleed Bin Talal Bin Abdulaziz Alsaud Institute for Computational Biomedicine, Weill Cornell Medicine, New York, NY 10021, USA; Department of Physiology and Biophysics, Weill Cornell Medicine, Cornell University, New York, NY 10021, USA; The HRH Prince Alwaleed Bin Talal Bin Abdulaziz Alsaud Institute for Computational Biomedicine, Weill Cornell Medicine, New York, NY 10021, USA; BioAstra Inc, New York, NY, USA; The Feil Family Brain and Mind Research Institute, Weill Cornell Medicine, NY 10021, USA; WorldQuant Initiative for Quantitative Prediction, Weill Cornell Medicine, New York, NY 10021, USA; Department of Physiology and Biophysics, Weill Cornell Medicine, Cornell University, New York, NY 10021, USA; Children's Cancer and Blood Foundation Laboratories, Departments of Pediatrics and Cell and Developmental Biology, Drukier Institute for Children's Health, Meyer Cancer Center, Weill Cornell Medicine, New York, NY 10021, USA; Meyer Cancer Center, Weill Cornell Medicine, New York, NY 10065, USA; Washington University St. Louis Oncology Division, St. Louis, MO 63100, USA; Washington University St. Louis Oncology Division, St. Louis, MO 63100, USA; Department of Physiology and Biophysics, Weill Cornell Medicine, Cornell University, New York, NY 10021, USA; Department of Physiology and Biophysics, Weill Cornell Medicine, Cornell University, New York, NY 10021, USA; The HRH Prince Alwaleed Bin Talal Bin Abdulaziz Alsaud Institute for Computational Biomedicine, Weill Cornell Medicine, New York, NY 10021, USA; Department of Physiology and Biophysics, Weill Cornell Medicine, Cornell University, New York, NY 10021, USA; Meinig School of Biomedical Engineering, Cornell University, Ithaca, NY 14853, USA; Meinig School of Biomedical Engineering, Cornell University, Ithaca, NY 14853, USA; Meinig School of Biomedical Engineering, Cornell University, Ithaca, NY 14853, USA; Department of Immunology, Weill Cornell Medicine, Cornell University, New York, NY 10021, USA; Department of Environmental and Radiological Health Sciences, Colorado State University, Fort Collins, CO 80523, USA; Sovaris Aerospace, Boulder, CO 80302, USA; Advanced Pattern Analysis & Human Performance Group, Boulder, CO 80302, USA; Sovaris Aerospace, Boulder, CO 80302, USA; Advanced Pattern Analysis & Human Performance Group, Boulder, CO 80302, USA; Element Biosciences, San Diego, CA 10055, USA; Element Biosciences, San Diego, CA 10055, USA; Element Biosciences, San Diego, CA 10055, USA; Element Biosciences, San Diego, CA 10055, USA; Element Biosciences, San Diego, CA 10055, USA; Element Biosciences, San Diego, CA 10055, USA; Element Biosciences, San Diego, CA 10055, USA; Element Biosciences, San Diego, CA 10055, USA; Department of Stem Cell Biology and Regenerative Medicine, Keck School of Medicine, University of Southern California, Los Angeles, CA 90033, USA; Department of Physiology and Biophysics, Weill Cornell Medicine, Cornell University, New York, NY 10021, USA; Department of Environmental and Radiological Health Sciences, Colorado State University, Fort Collins, CO 80523, USA; Cell and Molecular Biology Program, Colorado State University, Fort Collins, CO 80523, USA; Washington University St. Louis Oncology Division, St. Louis, MO 63100, USA; Space Exploration Technologies Corporation, Hawthorne, CA 90250, USA; Department of Physiology and Biophysics, Weill Cornell Medicine, Cornell University, New York, NY 10021, USA; The HRH Prince Alwaleed Bin Talal Bin Abdulaziz Alsaud Institute for Computational Biomedicine, Weill Cornell Medicine, New York, NY 10021, USA; BioAstra Inc, New York, NY, USA; The Feil Family Brain and Mind Research Institute, Weill Cornell Medicine, NY 10021, USA; WorldQuant Initiative for Quantitative Prediction, Weill Cornell Medicine, New York, NY 10021, USA

**Keywords:** genomes, clonal, hematopoiesis, stability, immune, mitochondria, ribosomes, spaceflight

## Abstract

**Background:**

The Inspiration4 (I4) mission, the first all-civilian orbital flight mission, investigated the physiological effects of short-duration spaceflight through a multi-omic approach. Despite advances, there remains much to learn about human adaptation to spaceflight's unique challenges, including microgravity, immune system perturbations, and radiation exposure.

**Methods:**

To provide a detailed genetics analysis of the mission, we collected dried blood spots pre-, during, and post-flight for DNA extraction. Telomere length was measured by quantitative PCR, while whole genome and cfDNA sequencing provided insight into genomic stability and immune adaptations. A robust bioinformatic pipeline was used for data analysis, including variant calling to assess mutational burden.

**Result:**

Telomere elongation occurred during spaceflight and shortened after return to Earth. Cell-free DNA analysis revealed increased immune cell signatures post-flight. No significant clonal hematopoiesis of indeterminate potential (CHIP) or whole-genome instability was observed. The long-term gene expression changes across immune cells suggested cellular adaptations to the space environment persisting months post-flight.

**Conclusion:**

Our findings provide valuable insights into the physiological consequences of short-duration spaceflight, with telomere dynamics and immune cell gene expression adapting to spaceflight and persisting after return to Earth. CHIP sequencing data will serve as a reference point for studying the early development of CHIP in astronauts, an understudied phenomenon as previous studies have focused on career astronauts. This study will serve as a reference point for future commercial and non-commercial spaceflight, low Earth orbit (LEO) missions, and deep-space exploration.

## Introduction

Human spaceflight frequency has increased over the last decade, and with it, our grasp of its effects on human physiology. Despite this, there is much to learn about how humans adapt to the unique challenges of the space environment. The space environment includes unique stressors, such as microgravity (e.g. weightlessness), immune system perturbations, and space radiation exposure. Determining the effects of spaceflight on the human genome is imperative to support long-term human presence in space. Additionally, a host of physiological adaptations occur when humans travel into space, ranging from cardiovascular and musculoskeletal deconditioning, vision changes (e.g. spaceflight associated neuro ocular syndrome), immune suppression, and metabolic changes, among others [1]. Moreover, increasing evidence highlights the systemic immune dysregulation crewmembers experience from spaceflight exposure. Indeed, there appears to be a reduction in T cell frequency, suppressed cytotoxic function, as well as fluctuations in cytokine concentrations, with increased expression of TNF-*α*, interleukin-8, interleukin-1ra, thrombopoietin, VEGF, and various chemokines (CCL2, CCL4, CXCL5, etc.) associated with spaceflight [2].

To date, almost all space missions have been led by professional astronauts from government space programs. More recently, however, SpaceX launched a crew of four civilian astronauts on the Inspiration4 (I4) mission, marking a new era for human space exploration. The present I4 mission study aims to investigate the impact of short-duration spaceflight on civilians, along with the post-flight and long-term biomedical, immunologic, and genetic alterations resulting from spaceflight. This includes rapid telomeric responses, whereby telomere elongation occurs as a function of spaceflight, followed by shortening upon return to Earth [3]. Both unusually short and long telomeres have been associated with adverse health effects involving aging and age-related pathologies, such as cardiovascular disease and cancer. Furthermore, a variety of lifestyle factors and environmental exposures influence telomere length. Previous studies have also shown changes in clonal hematopoiesis, where a small number of hematopoietic stem cells (HSCs) gain a clonal advantage through the acquisition of somatic mutations. Clonal hematopoiesis is associated with cardiovascular disease, the development of acute myeloid leukemia (AML), and increased overall mortality [4, 5]. The NASA Twins Study shed light on telomeric responses and the chronic inflammatory state resulting from long-duration spaceflight and provided the first measures of telomere length and clonal hematopoiesis in astronauts [3, 6]. Mutations in epigenetic regulators, such as DNMT3A and TET2, were found at increased rates in both career astronaut twins when compared to their civilian counterparts. A retrospective study of 14 astronauts who flew shuttle missions has also shown an elevated presence of genetic abnormalities in CHIP-driver genes in the astronaut population [3, 5, 7].

Here we investigated immune adaptations, telomere length dynamics, cell-free DNA release, genomic stability, single-cell transcriptomic analysis, and biochemical adaptations of the I4 crewmembers traveling into lower earth orbit over the course of 3 days, to determine the effects of short-duration spaceflight. It is imperative to study these adaptations in this context for future space missions, as prolonged space environment exposure could exponentially increase the rate of mutational burden [8], be it due to the inflammatory milieu resulting from spaceflight or the particular challenges of the space environment, such as microgravity and radiation exposure. Here, we leveraged the I4 mission to examine the development of genetic abnormalities in the astronauts by studying their physiological response to the demands of spaceflight and quantifying the genetic changes that are associated with spaceflight.

## Methods

### Informed consent and ethics

This study was completed following appropriate ethical guidelines according to the Declaration of Helsinki, ICHGCP, and local regulations, as applicable, from each potential subject or each subject's legally authorized representative prior to participating in the research study. The protocol, the ICF, other written material given to the patients, and any other relevant study documentation were submitted to and approved by the appropriate ethics committee. This study was conducted under a protocol reviewed and approved by the applicable ethics committees and investigations were undertaken by scientifically and medically qualified persons, where the benefits of the study were in proportion to the risks. All subjects were consented at an informed consent briefing (ICB) at SpaceX (Hawthorne, CA), and samples were collected and processed under the approval of the Institutional Review Board (IRB) at Weill Cornell Medicine, under Protocol 21–05023569. All crew members provided written informed consent for data and sample sharing.

I4 launched from Kennedy Space Center's Launch Complex 39A and traveled into Low-Earth Orbit across a three-day mission, reaching an orbital altitude of approximately 364 miles and ultimately splashed down into the Atlantic Ocean.

### Dried blood spot (DBS) pre-flight, in-flight, and post-flight sampling

Crew members warmed and massaged their fingertips to maximize blood flow. Fingertips were sterilized (BZK antiseptic towelette, Dynarex, Reorder No. 1303) and punctured using a contact-activated lancet (BD Biosciences, #366 593) or a 21-gauge needle (BD Biosciences, #305 167). Whatman 903 Protein Saver DBS cards (Cytiva, #10 534 612) were used to capture, transfer, and then store capillary blood with a desiccant pack (Cytiva, #10 548 239) at ambient temperature.

### DNA extraction for qPCR-based assessment of telomere length

Three 3 mm circular punches were cut from the Whatman 903 Protein Saver Cards (cat# WHA10534612) containing blood samples using an Integra Miltex Standard Biopsy Punch (cat# 12-460-406) and placed into a 1.5 mL microcentrifuge tube with sterile tweezers. Samples were prepared using the Qiagen QIAamp DNA Investigator Kit (cat# 56 504) following the manufacturer's isolation of total DNA from FTA and Guthrie Cards protocol. The quantification of DNA in each sample was determined through fluorometric quantification with the Qubit 4 Fluorometer (Thermo Fisher Scientific, cat# Q33238) and the 1X dsDNA HS Assay Kit (cat# Q33231). DNA samples were sent to Colorado State University for multiplex qPCR analysis.

### Multiplex quantitative PCR telomere length measurement

MMqPCR measurements of telomere length were carried out by preparing 22 μL of master mix using SYBR green GoTaq qPCR master mix (Promega #A6001) combined with the telomere forward primer (TelG; 5′-ACACTAAGGTTTGGGTTTGGGTTTGGGTTTGGGTTAGTGT-3′), telomere reverse primer (TelC; 5′-TGTTAGGTATCCCTATCCCTATCCCTATCCCTATCCCT AACA-3′), albumin forward primer (AlbU; 5′-CGGCGGCGGGCGGCGCGGGCTGGGCGGA AATGCTGCACAGAATCCTTG-3′), albumin reverse primer (AlbD; 5′-GCCCGGCCCGCCG 4 CGCCCGTCCCGCCGGAAAAGCATGGTCGCCTGTT-3′) at 10 μM per primer (Integrated DNA Technologies), and RNase/DNase free water. Then 3 μL of DNA at 3.33 ng/uL was added for a final volume of 25 μL, final TelG/C primers concentration of 900 nM, and the AlbU/D primers at 400 nM. A Bio-Rad CFX-96 qPCR machine was used to measure telomere length. The cycle design was as follows: 95°C for 3 min; 94°C for 15 s, 49°C for 15 s, for 2 cycles; 94°C for 15 s, 62°C for 10 s, 74°C for 15 s, 84°C for 10 s, and 88°C for 15 s, for 32 cycles. The melting curve was established by a 72°C to 95°C ramp at 0.5°C/second increase with a 30 second hold. Standard curves were prepared using human genomic DNA (Promega Cat # G3041) with 3-fold dilutions ranging from 50 ng to 0.617 ng in 3 μL per dilution. Negative controls included a no-template TelG/C only and AlbU/D only, and a combined TelG/C and AlbU/D control. Samples were normalized across plates using a human genomic DNA standard. Each sample was run in triplicate on a 96-well plate format and relative telomere length was established using a telomere (T) to albumin (A) ratio.

### Whole genome extraction and sequencing

Genomic DNA was obtained from the cell pellet of a cell-free DNA blood collection tube (Streck, cat# 230470) using the QIAamp Blood Maxi Kit (Qiagen, cat# 51192), and then shipped to Element Biosciences for library preparation. The extracted DNA was quantified using Thermo Fisher Qubit dsDNA HS Assay Kit (cat# Q238253) and 8 samples were prepared using the KAPA HyperPrep Kit and KAPA Unique-Dual Indexed Adapter Kit (cat# 8 861 919 702). The DNA libraries were quantified using Thermo Fisher Qubit dsDNA HS Assay Kit (cat# Q32854) and sized using Agilent High Sensitivity DNA Kit (cat# 5067–4626).

The 8 DNA libraries generated with the KAPA HyperPrep Kit were processed using Adept Library Compatibility Kit (Element Biosciences, Cat# 830–00003), individually circularized with 0.5pmol (30 μL of 16.67nM) input, and quantified using the kit-provided qPCR standard and primer mix. The libraries were pooled into 4 separate 2-plex pools, each denatured and sequenced on Element AVITI system (Element Biosciences, Part #88–00 001) using 2 × 150 paired end reads with indexing. Primary analysis was performed onboard the AVITI sequencing instrument.

### Whole genome/cfDNA preprocessing

Blood and plasma samples were subjected to whole genome and cfDNA short read sequencing as detailed above. Resultant FASTQ files were validated using FastQC (v0.11.9) and MultiQC (v1.13). Read adapters were trimmed at 3′ and 5′ ends for low quality using Trim Galore (v0.6.5), lower quality reads were classified and removed, retaining only those reads with length >= 25bp, and ph read quality >= 20. Reads were aligned against the hg38 human reference genome with BWA MEM (v.0.7.15) and subjected to standard QC and deduplication procedures as a part of Sentieon's TNscope (v202010) DNAseq workflow [9].

### Whole genome/cfDNA/single cell variant calling

Aligned and preprocessed reads were subjected to the TNScope variant calling pipeline. Calls were filtered using Fisher's exact test and subsetted to SNP variants using samtools (v1.16.1), and filtered by triallelic sites, short tandem repeats, read quality, and read position bias using BCFtools (v1.16). Varient-Effect-Predictor VEP (v107) was utilized for annotation of variants and further filtering based on predicted impact of mutations. Resulting coordinates were processed into allele and gene frequency matrices, and visualized in R using the tidyverse (v1.3.2) suite of packages.

### cfDNA extraction and sequencing

cfDNA was isolated from 500 μL aliquots of plasma from cfDNA blood collection tubes (Streck, #230470). cfDNA was extracted from each crew member from all timepoints (4 crew members, 6 timepoints, 24 total extractions). cfDNA was extracted using Qiagen's QIAamp ccf DNA/RNA Kit and eluted in 15 μL Qiagen Elution Buffer per sample. Yield was measured for each sample using Thermo Fisher Qubit 1X dsDNA HS Assay (cat# Q33231).

Entire extract volume was used as input for library preparation using NEBNext Ultra II DNA Library Preparation Kit for cfDNA protocol. Each sample was barcoded using NEBNext Multiplex Oligos for Illumina (Unique Dual Index UMI Adaptors–96 reactions). Final library was eluted in 30 μL and checked for concentration using Thermo Fisher Qubit 1X dsDNA HS Assay (cat# Q33231). Fragment sizes were determined using Agilent's Tapestation 2100 and D1000 reagents and ScreenTape, with resulting average fragment size ∼380 bp (0.25 pmol) of each sample.

A total of 24 cfDNA libraries generated with the NEBNext Ultra II DNA Library Preparation kit were processed using Adept Library Compatibility Kit (Element Biosciences, Cat# 830–00003). Each library was circularized individually with an input range of 0.2–0.5 pmol (30 μL of 6.67–16.67nM) based on linear library yields. The final circularized libraries were quantified using qPCR standard and primer mix and pooled into 2 separate 4-plex pools. Each 4-plex pool was denatured and sequenced on Element AVITI system (Element Biosciences, Part #88–00001) using 2 × 147 paired reads with 19 bp UMI/index 1 and 8 bp index 2. Primary analysis was performed onboard the AVITI sequencing instrument.

### Clonal hematopoiesis targeted variant calling

Genomic DNA was obtained from the cell pellet of a cell-free DNA blood collection tube (Streck, cat# 230 470) using the QIAamp Blood Maxi Kit (Qiagen, cat# 51 192). All samples from the testing and validation cohort were sequenced using a custom designed DNA sequencing assay (DB0188, VariantPlex, ArcherDX). This panel captures the nine genes most commonly mutated in solid tumor patients following therapeutic radiation including the full exonic regions of five genes (*DNMT3A, TET2, ASXL1, TP53, CHEK2*) and targeted exonic regions of four genes (*JAK2, SRSF2, SF3B1, PPM1D*). Libraries were prepared from 250 ng gDNA using the VariantPlex protocol (ArcherDx Inc., Boulder, CO, USA) which utilizes Anchored Multiplex PCR (AMP) technology to generate target-enriched sequencing-ready libraries. Following DNA fragmentation ligation with a universal ArcherDx molecular barcode (MBC) adapter is performed, which tags each DNA molecule with a unique molecular index (UMI) and allows for unidirectional amplification of the sample using gene-specific primers. The resulting libraries were sequenced using a NovaSeq 6000 instrument (Ilumina), as per manufacturer's instructions. The methods for variant identification are comparable to those in Novetsky et al. *2023* [10]. The variant calling pipeline included UMI consensus building followed by utilizing Mutect2, VarDictJava, Lofreq2, Pindel in parallel for variant identification. Normalization was performed via LeftTrimAndAlign, and a panel of normals of 27 children and young adults was used with a bonferroni-corrected p value to identify true putative mutations in our astronaut cohort. A pileup analysis as performed in Bolton et al. *2020* [11] was used to query variants in longitudinal follow-ups. A variant list was used to scrutinize raw BAM files and the PoN samples, a fisher's exact test and Bonferroni-corrected p value were used to determine a noise threshold. Variants surpassing this threshold in at least one sample were further examined in the remaining samples, and if statistically validated, the variants were aggregated to construct the final variant list. Variant-Effect-Predictor VEP (v107) and SnpEff (v4.3) was used for annotation of variants. Data wrangling, tidying, and visualizations were performed using R (v4.1.2), Rstudio (v2021.09.2) and libraries (Tidyverse, Dplyr, data.table, ggplot2).

### cfDNA fragment analysis

Fragment size distribution was calculated using the bamPEFragmentSize tool from the deepTools Python package (v3.5.1). Levels of cfDNA (read counts) originating from different chromosomes were normalized by chromosome length and total number of reads in the library generating a Read per Kilobase per Million reads (RPKM) measurement. The fraction of cell-free mtDNA relative to chromosomal cfDNA in plasma was compared and visualized in R using the tidyverse (v1.3.2) suite of packages.

### cfDNA tissue of origin deconvolution

The enrichment of cfDNA fragments from various tissues was calculated by read coverage depletion analysis at transcription starting sites (TSSs) to estimate nucleosome positioning and infer gene expression. The pipeline is described in detail in Bezdan et al. 2020 [7]. The resulting nucleosome periodicity was correlated with (1) per-tissue gene expression reference matrix retrieved from the Human Proteome Map (HPM; Kim et al. 2014 [12]) or (2) individual astronaut pseudo-bulk expression of different cell subpopulations extracted from PBMC scRNAseq dataset. In both cases the tissue/subpopulation-periodicity correlations were ranked by the value of Pearson's correlation coefficient, clustered (utilizing Ward method with Euclidean distances) and visualized in R using the tidyverse (v1.3.2) and ComplexHeatmap (v2.14.0) packages.

### Single-cell gene expression analyses

Longitudinal single cell data was processed in R using the Seurat package (v4.3.0) to normalize, scale and cluster cell populations. Cell identities were determined through computational gating parameters of inclusion based on gene expression of key markers, similar to gating from Fluorescent-Activated Cell Sorting. CD8+ T cells were selected from the PBMC population by filtering CD3D+ CD8A+ positive cells. CD4+ T cells from CD3D+ CD8A+ cells, CD14+ monocytes from CCR2+ CD14+ cells, CD16+ monocytes from CD14+ CD16+ cells, NK cells from NCAM1+ CD3- and NCR3+ CD3- cells and DCs from CD86+, CD83+ cells.

The Seurat package (v4.3.0) was used for normalization, scaling, and differential gene expression analysis of sn-RNA data. Data wrangling, tidying, and visualizations were performed using R (v4.1.2), RStudio (v2021.09.2) and libraries (Tidyverse, Dplyr, data.table, ggplot2).

## Results

### Telomere length measurement (MMqPCR)

**Figure 1.**
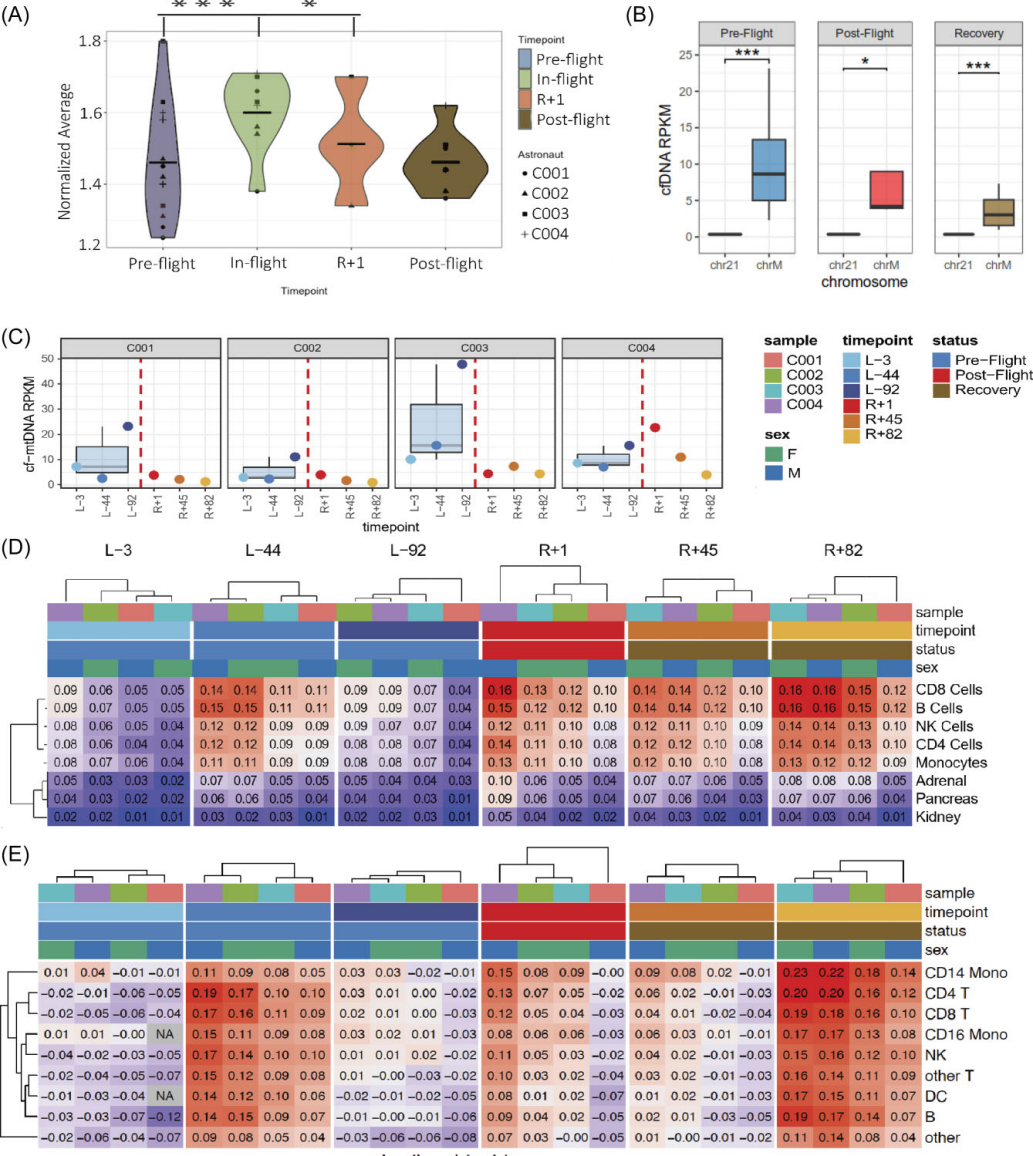
Telomere length dynamics, mitochondrial, and cell-free DNA cell lysis Identity as a function of spaceflight. (A) Telomere length dynamics assessed by MMqPCR in I4 crew members. Normalized analysis shows increased average telomere length during orbital flight compared to pre-flight baseline and in R + 1 compared to pre-flight through Tukey-corrected repeated measures ANOVA (*P *< 0.001) and (*P *< 0.02), respectively. A rapid decrease in telomere length was also seen after landing during post-flight recovery months. (B) Autosomes do not show any spaceflight-related change in RPKM (reads per kilobase per million reads), as exemplified using chr21. The cf-mtDNA is significantly more enriched in all samples—significance levels (*P*-value) of the Wilcoxon test are as follows: Pre-Flight < 0.001, Post-Flight = 0.029, Recovery < 0.001. (C) Observed cf-mtDNA levels show high inter-sample heterogeneity. (D) Tissue of origin deconvolution for circulating cfDNA fragments reveals an increased cfDNA signature of both adaptive and innate immune cells post-landing and during recovery. The enrichment of tissue signatures from the HPM [13] was calculated based on inferred gene expression and nucleosomal footprinting of the cfDNA fragments. Average correlation coefficients (multiplied by -1) over technical replicates are depicted for each sample and time point. The heatmap was subsetted to relevant tissue signatures; extended data is available in Supplememtary Fig. S1 and Table S1, see online supplementary material. (E) Cross-examination of cfDNA origin deconvolution using each of I4 astronaut cell-subpopulation-specific expression markers, derived from peripheral blood (PBMC) dataset, supports the results in D. We note an increased presence of innate and adaptive immune cfDNA in R + 1 and R + 82 when compared to all other timepoints. The extended data is available in Supplememtary Tables S2, see online supplementary material.

We first examined DNA from dried blood spots (DBS), which were collected from the I4 crew members before (L-92, L-44, L-3), during (FD1, FD2, FD3), and after (R + 1, R + 45, R + 82) spaceflight, from which DNA was isolated, and telomere length measured via multiplexed quantitative PCR (MMqPCR) (Fig. 1A). Telomere elongation was observed in all four I4 crewmembers during spaceflight, as compared to their pre- and post-flight normalized means (Fig. 1A). Telomere length shortened rapidly upon return to Earth in 3 of the 4 astronauts (post-flight), and overall continued to shorten over the course of the recovery period. Repeated-measurements ANOVA was performed on the data to assess the overall impact of time on telomere length. Post hoc pairwise comparisons identified pre-flight vs in-flight (*P *< 0.001) and pre-flight vs R + 1 (*P *< 0.02) as timepoints between which significant differences in telomere length occurred.

Intriguingly, despite the short duration of the I4 mission (3 days of orbital flight), these results correlated with findings from the NASA Twins Study (One Year Mission astronaut), as well as with astronauts on ∼6 month missions onboard the International Space Station (ISS) [3, 13].

### Cell-free DNA

Next, cell-free DNA (cfDNA) from the I4 crew's plasma was extracted from Streck Cell-free DNA BCT tubes and sequenced to compare to pre-flight baseline, post-flight, and recovery period responses. Extraction and pre-processing of plasma were not implemented during the flight, so all samples were collected on Earth to mitigate cfDNA contamination from apoptotic blood cells during sample transportation (Supplememtary Fig. S1A & B, see online supplementary material) [8]. We analyzed plasma mitochondrial cfDNA (cf-mtDNA) relative to the chromosomal cfDNA fraction (Fig. 1B & C, Supplememtary Table S3, see online supplementary material) as a potential biomarker for long-duration spaceflight adaptations. In this mission, cf-mtDNA levels did not rise significantly following the return to Earth (R + 1), possibly due to the short flight duration. Cf-mtDNA enrichment was only observed in the twin astronauts on the ISS after months of exposure to the space environment. The marker levels display high between-sample heterogeneity, and the increase in pre-flight measurements may be connected to the I4 crew's pre-flight preparation (e.g. high-altitude training).

Given that cfDNA fragments encompass one nucleosome (∼150 bp), we inspected depletion in read coverage distribution around the transcription start sites (TSS) as a measurement of gene expression [6, 14]. We applied coverage normalization specific to the genomic neighborhood and completed Fourier transformation to determine nucleosome footprints based on signal periodicity [7]. The cfDNA fragment tissue and cell-type-of-origin enrichment of fragments were correlated with either tissue-specific expression signatures from the Human Proteome Map (HPM) [12] or individual astronaut expression profiles from peripheral blood [15] (Fig. 1D & E, Supplememtary Fig. S1C, Supplememtary Tables S1 & S2, see online supplementary material). The most represented sequences were of hematopoietic origin, while a small increased presence of cfDNA fragments from both innate and adaptive immune cells was observed post-landing and during the recovery period. Interestingly, there was a significant increase in the cfDNA originating from all immune cells in all astronauts over a month following their return to Earth (R + 82), indicating a degree of heterogeneity in cell lysis over time and a possible long-term response.

### Clonal hematopoiesis of indeterminate potential (CHIP) assessment

**Figure 2.**
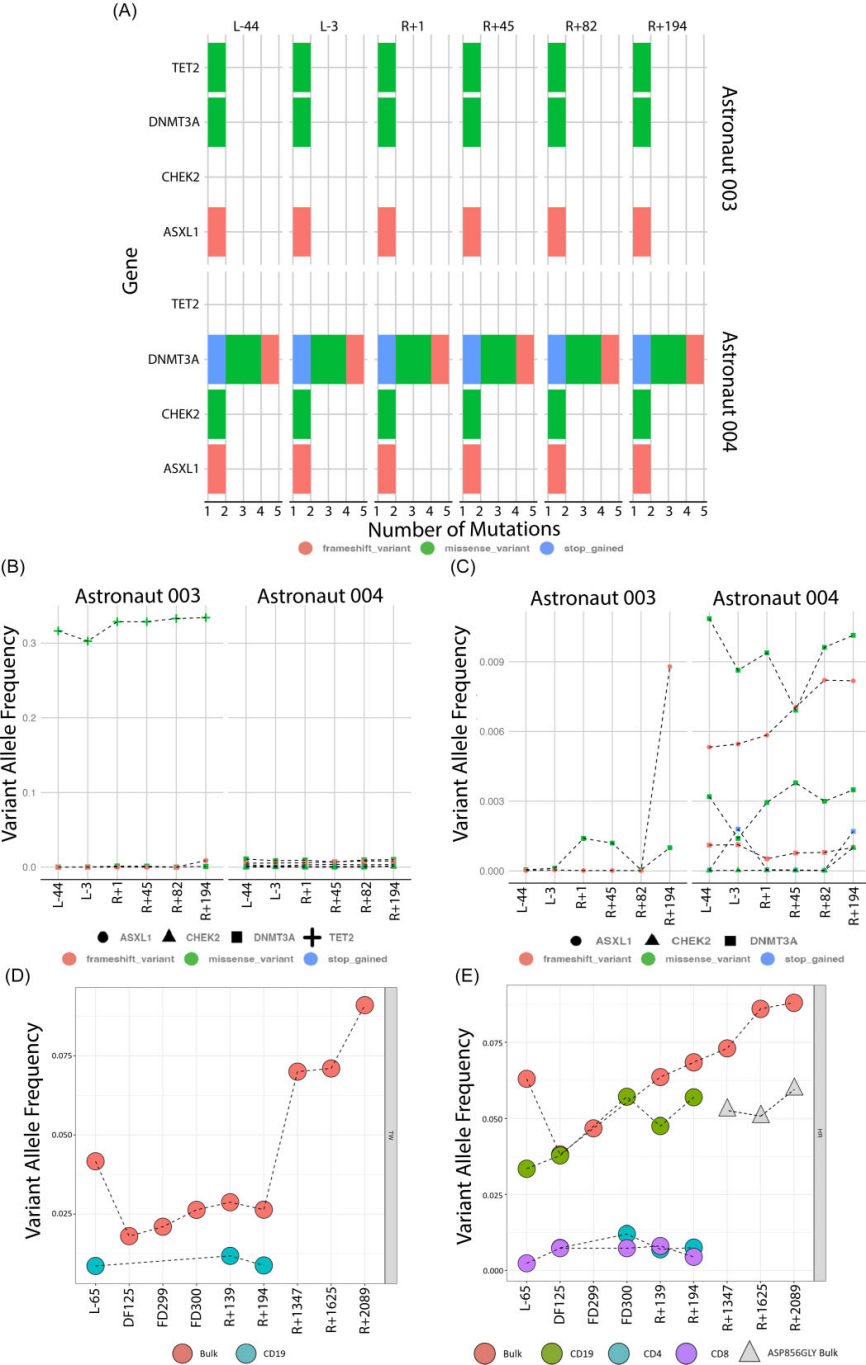
Targeted deep sequencing of CHIP-related genes exhibit genomic stability and comparable mutational burden as a function of spaceflight. (A) Putative driver mutational burden of CHIP-related genes were found in two out of the four astronauts, longitudinal follow-ups demonstrate stability during the months leading and following spaceflight. (B&C) Variant allele frequency of putative driver mutations (chr2: g.25235778C > G, chr2: g.25234347G > C, chr2: g.25241591C > A, chr2: g.25246732GTCGTGGCACACCGGGAACAGCTTCCCCGC > G, chr2: g.25247647G > A, chr22: g.28734438C > T, chr20: g.32434638AG > A, chr4: g.105275662G > T) in subjects 003 and 004 remained comparable both during pre-flight timepoints and post-flight recovery. (D&E) Comparison of variant allele frequency of TET2.p.Cys1273Tyr mutation in TW (spaceflight) subject of the NASA Twins Study and variant allele frequency of DNMT3A.p.Trp698Ter and de-novo mutation DNMT3A.p.Asp856Gly in HR (ground subject) showed a relative stable VAF during the initial stages of the study but an increased variant allele frequency through a 6 year follow-up period.

While CHIP has been previously studied in astronauts, the I4 cohort provided an opportunity to study the physiological impact of spaceflight in a civilian crew that had never undergone orbital flight. Indeed, the monitoring of this crew provides us with a unique opportunity to understand the beginning of the already established relationship between career astronauts and CHIP. Of note, the NASA Twins Study assessed the mutational burden of long-duration spaceflight in CHIP genes and a retrospective study established a burden of CH-related mutations in the career astronaut population [3, 4] and we sought to examine these same CHIP genes for the I4 crew. To address the variant allele frequency (VAF) of CHIP genes, we performed deep targeted sequencing (>15,000x) of 10 known epigenetic regulators and CHIP-associated genes and assessed the mutational burden of short duration spaceflight in the I4 astronaut cohort. Notably, the mutational burden of the CHIP-associated genes did not change as a function of spaceflight and remained stable for up to 6 months post spaceflight (Fig. 2A). No known deleterious mutations arose in the I4 cohort post-flight, nor did any pre-existing somatic mutations increase in allele frequency significantly (Fig. 2B & C). Putative driver mutations were found in two out of the four astronauts but their VAF remained comparable at all timepoints, indicating stability of the VAF from the spaceflight.

To give context for these data, we next collected additional samples for both subjects in the NASA Twins Study. Subject TW (long duration spaceflight) presented mutation TET2.p.Cys1273Tyr in a bulk PBMC sample at an increased VAF of ∼ 0.075 (Fig. 2D). Subject HR (ground control) presents the mutation DNMT3A.p.Trp698Ter at an increased VAF of ∼ 0.086 and the mutation DNMT3A.p.Asp856Gly not previously found at a VAF of ∼ 0.05 (Fig. 2E). Both astronauts presented increased clonality when compared to previous timepoints. Their mutational burden was still greater than age-matched controls, as established in the original study. However, the increased mutational burden is an expected finding, given the association between aging and CHIP [16] and there's no evidence of them having any clinical effects to date.

### Longitudinal comparison of whole genome mutations reveals genomic stability months after short-duration orbital flight

**Figure 3.**
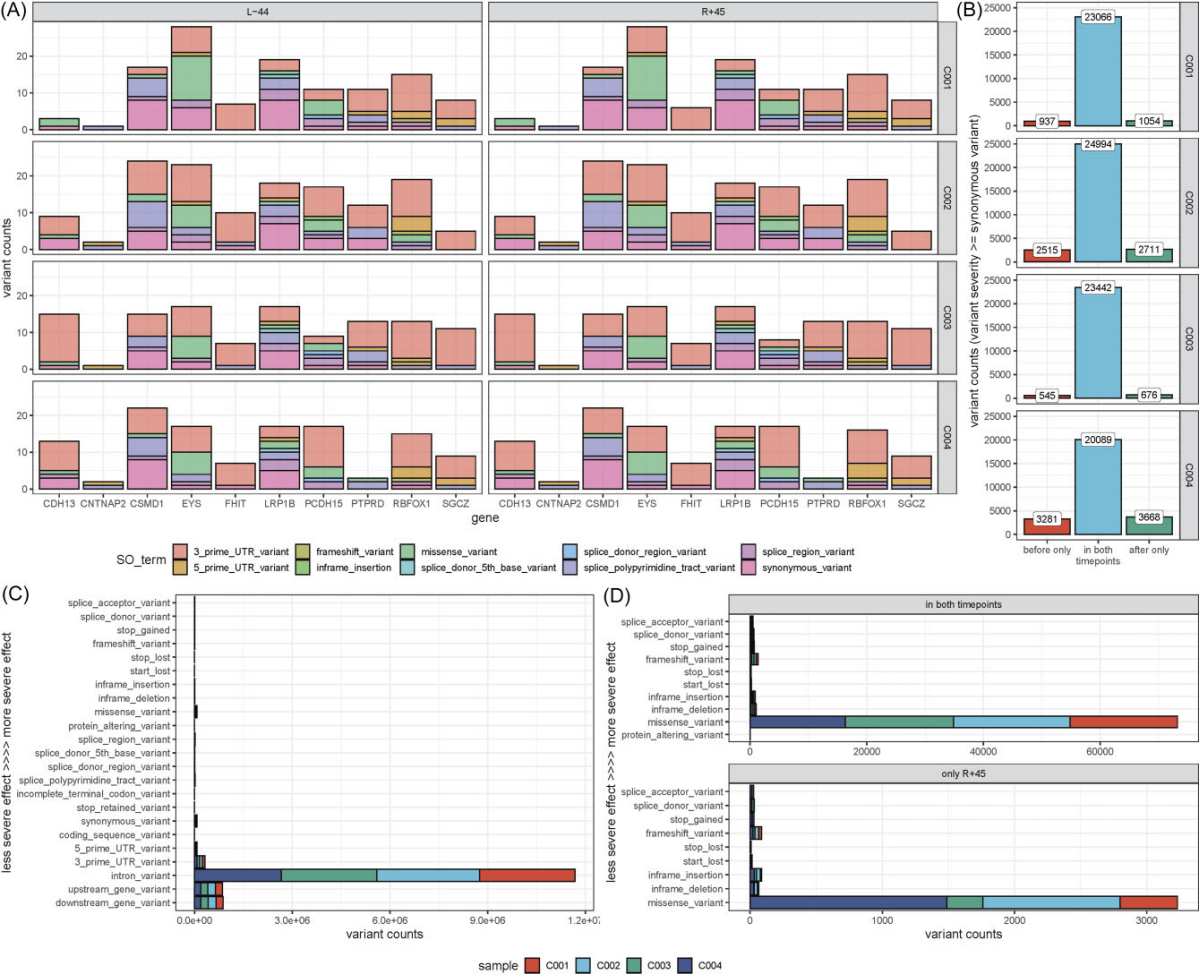
Whole genome sequencing and variant calling reveals genomic stability post-flight. (A) Comparison of variant consequences before and after spaceflight of genes with the greatest mutational burden demonstrates no gene is disproportionately mutated post-flight. We report unique counts of (variant, effect annotation) pairs per gene of interest. (B) De novo mutational comparison elucidates comparable mutational burdens at a genome-wide scale. Here we count variants as unique changes of reference to alternative alleles at a given position regardless of variant annotation. (C) Variant effect annotation for variants called in both timepoints shows most mutations are in non-coding regions. Variant annotations were ranked according to the severity of the variant effect estimated by Ensembl [15]. We report unique counts of (variant, effect annotation) pairs across the whole genome. (D) Timepoint comparison of variant effect annotations across the whole genome shows no disproportional mutational load for variants called uniquely post-flight. Here we show the distribution for moderate to high-severity variants.

To assess the long-term consequences of spaceflight on the genomic stability of astronauts, we conducted whole-genome sequencing (WGS) and variant calling pre- and post-flight. Comparison of single nucleotide variant (SNV) and indel presence between timepoints (L-44 and R + 45) for each astronaut indicated no significant changes for genes with the highest mutational burden (Fig. 3A), epigenetic regulator genes (Supplememtary Fig. S3, see online supplementary material) or other gene sets of interest. The analysis of variant annotations (Fig. 3A & C) revealed that most identified variants are within intronic regions and have no significant effects. We also have noted no disproportional mutational load for variants post-flight (Fig. 3D). We performed variant calling in single nucleus RNA-seq (snRNA-seq) libraries and compared it across all timepoints and against WGS for genes with read coverage > 10x (Supplememtary Fig. S3B & C, see online supplementary material). Whole genome coverage of the snRNA-seq libraries varied broadly across timepoints, but did not show any significant evidence of genomic instability within our cohort.

### Spaceflight induces cell-type-specific changes in gene expression profiles

We next examined gene expression changes across immune cell types due to spaceflight. Cell type-specific genes were used to filter cell types computationally. CD8+ T cells, and CD4+ T cells, CD14+ monocytes, CD16+ monocytes, dendritic cells (DCs) and natural killer (NK) cells were filtered from PBMCs at six different timepoints: L-92, L-44, L-3, R + 1, R + 45, and R + 82.

**Figure 4.**
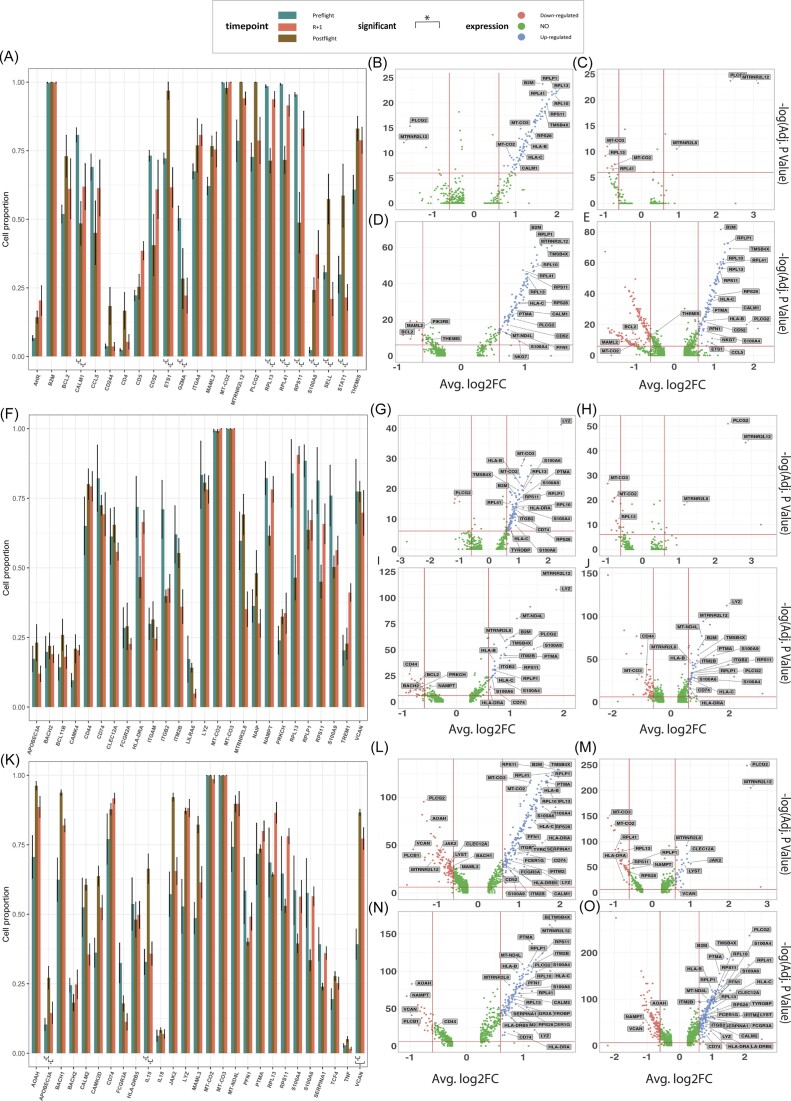
Differential gene expression and frequency of specific immune subsets reveals conserved, long and short-term adaptations across adaptive and innate immune cells. (A) Frequency of CD8 T cells expressing genes. (B-E) CD8 T cell volcano plot comparison of differential gene expression in L-92 vs R + 1, L-92 vs R + 82 & R + 1 vs R + 82. (F) Proportion of classical monocytes expressing genes. (G-J) Classical monocytes longitudinal differential expression in L-92 vs R+1, L-92 vs R+82, R+1 vs R+82 & R+1 vs all timepoints. (K) Proportion of dendritic cells expressing genes. (L-O) Dendritic cells longitudinal differential expression in L-92 vs R+1, L-92 vs R+82, R+1 vs R+82 & R+1 vs all timepoints.

Gene expression of CD8+ T cells at R + 1 and R + 82 showed increased expression of genes related to immune function, ribosomal and mitochondrial activity (e.g. *NKG7, CCL5, HLA-C, S100A4, RPL41, RPS11, MT-CO3, PTMA, B2M*, see Fig. 4B). Similar trends were seen with CD14+ monocytes, DCs, CD4+, FCGR3A+ monocytes, NK cells, and CD3D+ cells (Supplememtary Fig. S4C & D, see online supplementary material). Increased gene expression of mitochondrial and ribosomal genes was conserved across cell types for both L-92 vs R + 1 and L-92 vs R + 82 comparisons. PLCG2, MTRNR2L12, VCAN, and MAML2 transcripts associated with immune function, mitochondrial function, cell adhesion, and Notch signaling were consistently downregulated across all cell types (Fig. 4 & Supplememtary Fig. S4).

Interestingly, the proportion of cells expressing these genes does not necessarily change in parallel with total gene expression levels. For example, *PLCG2* expression, on a per cell basis, decreased, but the total proportion of cells expressing *PLCG2* increased among CD8+ T cells at R + 1 (0.98) vs. L-92 (0.81 +/- 0.07 s.e.) and R + 1 vs. R + 82 (0.66 +/- 0.04 s.e.), classical monocytes at R + 1 (0.99) vs. L-92 (0.96 +/- 0.01 s.e.) and R + 1 vs. R + 82 (0.92 +/- 0.05 s.e.) as well as DCs at R + 1 (0.99) vs. L-92 (0.96 +/- 0.01 s.e.) and R + 1 vs. R + 82 (0.92 +/- 0.04 s.e.) (Fig. 4A, C & E). Gene expression of mitochondrial cytochrome c oxidase, particularly subunits 2 and 3 (*MT-CO2* & *MT-CO3*), increased across all cell types, per cell, for R + 1 compared to L-92 and R + 82, with no significant change in the proportion of cells expressing these genes. *MTRNR2L12* expression was consistently decreased across all studied cell types for R + 1 vs. L-92 but greatly increased for R + 82 vs. L-92 and R + 82 vs. R + 1, suggesting changes in gene expression lasting over two months post-flight in both adaptive and innate immune cells.

## Discussion

The I4 mission is the first study to date to analyze the effect of short duration spaceflight on civilians using a comprehensive multi-omics approach. Our findings, including telomere length dynamics, CHIP-related clonal expansion, WGS genomic stability, cfDNA cell lysis analysis, and immune cell longitudinal gene expression profiling, broadly contextualize the physiological burdens of short duration spaceflight. This study adds key CHIP and genetic data on astronauts, which are limited, and can serve as a reference for future spaceflight planning, short missions around Earth's orbit, and longer-duration missions into deep space.

The NASA Twins Study was the first to report spaceflight-specific telomere elongation in humans, with one astronaut experiencing telomere elongation during his one-year mission, rapid telomere shortening upon return to Earth, and telomere length recovery to near pre-flight baseline values over the following months, although many more short telomeres after spaceflight than before were also observed [3]. Luxton et al. reported similar spaceflight-associated telomere length dynamics in 3 unrelated astronauts on ∼ 6-month missions onboard the ISS [13]. It was hypothesized that such dramatic shifts in telomere length dynamics were associated with chronic exposure to the space radiation environment and represented an adaptive response to chronic oxidative damage, specifically to telomeres, whereby the alternative lengthening of telomeres (ALT) pathway is transiently activated in normal somatic cells [13]. Cytogenetic evidence of heterogeneous telomere lengths and DNA damage responses were also reported, and—together with our cfDNA enrichment analyses—suggested increased DNA damage and are consistent with space radiation exposure and increased senescence-associated foci. Similar changes in telomere length dynamics observed in the I4 crew as a function of spaceflight indicate that telomere length alterations are fast-acting as associated with spaceflight, even for missions of short (e.g. days) duration. Circulating cell-free DNA (cfDNA) fragments, originating from various tissues and the immune system, offer a non-invasive method for assessing astronauts' dynamic immune responses to spaceflight-induced physiological stress. As an emerging biomarker, cfDNA concentration and molecular profile yield valuable insights into stress responses in unique spaceflight environments despite its heterogeneous and sensitive nature.

Our study indicates that the impact of short-duration spaceflight on cfDNA concentration in astronaut plasma is subtler than long duration missions, but increased innate and adaptive immune system activity is still apparent. Moreover, cfDNA enrichment from all immune cells persists post-flight, suggesting a delayed response involving immune cell turnover and active DNA repair mechanisms. This phenomenon warrants further investigation as it is imperative for both short and long duration spaceflight to elucidate the temporal changes to immune cell types in order to better predict and prevent future risks.

### Effects of short and long duration spaceflight on CHIP genomic alterations in civilian and career astronauts

In the I4 cohort, we found no relationship between spaceflight and increased CHIP-related genetic abnormalities in the scale of three months after spaceflight. With our deep sequencing targeted probe approach (Mean Depth ∼15,000x), we found putative mutations present in subject C004’s and C003 targeted samples but not C001 and C002. DNMT3A gene mutations chr2: g.25235778C > G, chr2: g.25234347G > C, chr2: g.25241591C > A are all missense variants with a likely low/moderate impact on function. A frameshift mutation, chr2: g.25246732GTCGTGGCACACCGGGAACAGCTTCCCCGC > G, was detected in astronaut 004, as well as a stop-gain function mutation chr2: g.25247647G > A. Although DNMT3A presented the higher number of mutations, CHEK2 presented a missense mutation chr22: g.28734438C > T in C004, and ASXL1 presented a frameshift mutation chr20: g.32434638AG > A in both C003 and C004. However, with a VAF of < 0.02 for all timepoints, although detectable, there is no evidence to suggest a clinical effect on the subjects, that is, spaceflight having no discernible effects up to 3 months post-flight. TET2 exhibited a missense mutation chr4: g.105275662G > T at a VAF ∼ 0.3 in C003. However, putative mutations in CH-linked genes were not found at disproportionate allele frequencies post-flight, nor were *de novo* mutations seen in the post-flight samples.

Although our findings suggest that no pathological genomic alterations occurred as a function of spaceflight in the I4 cohort, further longitudinal tracking of the astronauts will help us discern the long-term effects, if any, of short duration missions. Previous studies of the relationship between clonal hematopoiesis and astronauts has elucidated that spaceflight increases proportion of CH abnormalities in subjects when compared to healthy controls [3, 4]. It is not clear why that is the case, or when in an astronaut's career these abnormalities begin to appear, a key limitation of studying career astronauts. Previous studies have also shown the influence of acute events on the development of CH abnormalities, such as first responders from the world trade center tragedy [17]. Therefore, the I4 mission is uniquely positioned to characterize the clonal dynamics associated with a short-duration flight, and will allow future comparisons between career astronaut CH abnormalities and civilian space travelers. There is an opportunity to study the compound effects of spaceflight between missions on CH abnormalities if one or more of the I4 astronauts returns to space. This can be leveraged for a better understanding of long-term risk and clonal evolution.

The space environment is known to be chronically inflammatory as astronauts in previous studies have reported increased inflammation markers with the flight duration [18–20]. In the NASA Twins Study, both career astronauts had increased mutational burden in epigenetic regulators such as DNMT3A and TET2 compared to prostate cancer patient controls. Although only one of the twins was in orbital spaceflight for a year, both twins displayed comparable CHIP-related mutations shortly after flight and for several years of follow-up [6]. We continue to monitor the CHIP-related mutational burden of both astronauts years post-flight. The VAF of mutations found in both subjects has, as expected, increased over time, and a new missense variant has been identified in subject HR in the last 3-year follow-up. This is to be expected as CHIP-mutational burden is known to increase as a function of aging. Both astronauts, however, presented clonal burdens that preceded their age-matched control for over two decades at the time of the original study. The fact that TW (space subject) did not present a greater mutational burden post-flight than HR (ground control), despite the length of his last mission, might indicate that the overall number of flights, rather than their duration, is a greater extrinsic factor for clonal positive selection. The I4 cohort provides a unique opportunity to assess the short and long term physiological effects of a singular spaceflight mission. As civilians, the I4 cohort's physiological findings are likely a better representation of what first-time fliers may expect as we gear up toward an increased human presence in space.

Moreover, the longitudinal comparison of point mutations and indels from whole-genome sequencing data supports the results of the targeted deep-sequencing CHIP panel, wherein there is not a significant increase in the genome-wide mutational burden for first-flight civilian astronauts. These results favorably indicate that short-time spaceflight does not contribute to overall increased genomic instability, at least on a timescale of several months post-flight. Extending the analysis to future missions and continuous longitudinal tracking for years post-flight could shed more light on the long-term consequences of spaceflight in relation to its altitude and duration, or for low VAF alleles, as well as repeated exposure of astronauts to space radiation.

Finally, we note that transient changes in mitochondrial gene expression were seen as a response to spaceflight across a broad range of immune cells within the I4 cohort. Longitudinal gene expression analyses revealed a conserved trend across both adaptive and innate immune cells that had not been described before in the spaceflight literature. In comparisons between L-92 vs R + 1 and R + 1 vs R + 82 all immune cell types demonstrated increased expression of *MTRNRL12, MTRNR2L8*, and *MT-ND4L* in R + 1. *MTRNRL12* and *MTRNR2L8* are pseudogenes thought to be involved in negative regulation of the execution phase of apoptosis [21]. As such, increased *MTRNRL12 & MTRNR2L8* expression could indicate a physiological attempt at regulating increased immune cell death. Increased immune cell apoptosis was seen in our cfDNA analysis, at all post-flight timepoints, providing support to the gene expression alterations seen.

Of note, *MT-CO2 and MT-CO3* gene expression were downregulated post-flight (R + 1) compared to pre-flight (L-92) within CD86+ DCs, NCAM+ NK cells, CD14+ monocytes, and CD3 delta+ CD8+ & CD4+ T cells (Fig. 4B, D, F & supplememtary Fig. S4B, D, F, H). *MT-CO2* and *MT-CO3* encode subunits of cytochrome c oxidase, which is involved in the reduction of oxygen to water. Decreases in *MT-CO2* and *MT-CO3* expression cause significant cytochrome c oxidase and mitochondrial complex IV deficiencies, which may lead to tissue maladaptations [22, 23]. Most mitochondrial genes return to basal levels of expression in our L-92 vs R + 82 comparison, suggesting relief from spaceflight-induced mitochondrial oxidative stress. DCs, monocytes, and T cells showed increased *MT-ND4L* expression over two months after return from flight. The *MT-ND4L* gene encodes for NADH-ubiquinone oxidoreductase chain 4L, a component of the respiratory chain Complex I, a protein required for electron transfer and dehydrogenation from NADH to ubiquinone. Longitudinal changes in its gene expression may suggest that mitochondrial ATP production is altered long after return to earth. However, whether the gene expression is representative of decreased activity of the electron transport chain or if it is a physiological response seeking to restore mitochondrial function due to the hypofunctional aberrant effects of oxidative stress, remains to be elucidated.

In the spaceflight literature, human induced pluripotent stem cell-derived cardiomyocytes had increased expression of genes related to the mitochondrial electron transport chain, mitochondrial transit peptide, mitochondrial translocation, and mitoribosomes [24]. The NASA Twins Study also revealed transient enrichment of mitochondrial signaling that returned to baseline in the recovery period [25]. Our findings, in contrast, reveal a more complex picture of mitochondrial gene expression changes where some are consistently upregulated across cell types and others are transiently downregulated in R + 1 but return to basal levels in R + 82.

### Immune adaptations to short duration spaceflight

Immune system dysregulation in crewmembers following spaceflight has been consistently reported, revealing adaptive immune system changes and general shifts towards a Th2 T cell phenotype [26, 27]. Space environment exposure leads to suppressed activation and reduction in T cells, along with fluctuations in cytokine gene expression, such as increased TNF-a, interleukin-8, interleukin-1ra, thrombopoietin, VEGF, and various chemokines [2, 27, 28]. Our PBMC phenotype analysis offers insight into alterations within immune cell subpopulations, cell state, and genotype associated with short-duration orbital flight in an all-civilian crew. PLCG2 is known for its role in immune cell signaling by cleaving PIP2 into IP3 and diacylglycerol (DAG), key second messenger molecules that are involved in immune and growth factor receptors [29]. Additionally, although the exact physiological pathway is not known, PLCG2 has been found to positively affect mitochondrial respiration [30]. Dendritic cells exhibit increased *AHR* expression in R + 1 vs L-92 (Fig. 4, Supplememtary Fig. S4C), a transcription factor known to promote the development of pro-inflammatory Th17 and Th22 cells [31, 32]. T-helper (CD4+) populations present decreased *IL-32* expression (Supplememtary Fig. S4D) in R + 82 time points relative to pre-flight L-92, suggesting the transient changes to adaptive immune function can last up to two months, a finding concurrent with the body of literature of spaceflight research [33].

Immune dysfunction involving cytotoxic T cells is a proposed side effect of space travel [34], associated with an elevated risk of opportunistic infections during spaceflight, such as latent herpes virus reactivation [35]. Short-term exposure to spaceflight conditions may affect innate immune system components, including functional alterations of key immune cell populations like CD14+ monocytes and dendritic cells. Microgravity-induced changes can result in decreased neutrophil and macrophage phagocytic activity, thus affecting the initial response to pathogens. Moreover, microgravity may alter monocyte maturation and function, which play a critical role in antigen presentation and T cell activation. Kaur et al. (2005) describe that, although the percentage of CD14+ monocytes was not significantly reduced in astronauts following spaceflight, days after landing, the ability of these cells to engulf pathogens, degranulate, and mount an immune response was reduced compared to that of ground controls [36]. Though little is known about the influence of spaceflight on dendritic cell function, their role in inflammation is well characterized as they play key roles in modulating cytokine production, mediating migration, and enhancing antigen capture and processing [37].

Finally, while the long-term effects of short-duration spaceflight on the immune system and the genome are minimally understood, particularly in civilians and first-fliers, these data are critical as we expand human presence in orbit and deep space. Additional studies are needed across varying cell types, mission duration, and crew background, which can also help guide countermeasures.

Of note, the short-duration space shuttle studies revealed decreased lymphocyte response, post-flight neutrophil increase, and eosinophil decrease, correlating with in-flight stress rather than microgravity [28, 38, 39]. Latent herpesvirus reactivation and cytomegalovirus shedding were also observed during space shuttle flights [18]. Similar adaptations occurred during ISS missions, as crew members experienced mild infectious diseases, atypical allergies, or dermatitis without significant operational impact [18, 32], which seemed to be a function of mission duration. A 12-year ISS immune data comparison indicated improvements in immunity, stress, and viral reactivation due to operational and biomedical countermeasures onboard ISS, such as resupply frequency, improvements in personal communication, exercise equipment, and food quality and variety, suggesting the potential for improved quality of life on the health of astronauts [40]. Such operational and biomedical considerations from earlier missions, as well as the cellular and molecular data shown here, can help future crews and missions maintain crew health and safety, as well as guide efforts for lifetime health studies of astronauts.

## Conflict of interest

Christopher E. Mason is a co-Founder of Cosmica Biosciences. Braden Tierney is compensated for consulting with Seed Health and Enzymetrics Biosciences on microbiome study design and holds an ownership stake in the former. Kelly Bolton receives research funding from Servier and Bristol Myers Squibb and serves on the medical advisory board of GoodCell. Kelly Blease, Juan Moreno, Andrew Boddicker, Junhua Zhao, Bryan Lajoie, Andrew Altomare, Semyon Kruglyak, and Shawn Levy are employees of and have a financial interest in Element Biosciences. Irina Matei is a Co-Principal Investigator for research projects funded by Atossa Therapeutics. Caleb M. Schmidt, Julian C. Schmidt, and Michael A. Schmidt hold shares in Sovaris Holdings, LLC. Min Yu is the founder and president of CanTraCer Biosciences Inc. Authors not listed here do not have competing interests. Besides, as an Editorial Board Member of *Precision Clinical Medicine*, the corresponding author Christopher E. Mason was blinded from reviewing and making decision on this manuscript.

## Supplementary Material

pbae007_Supplemental_Files

## Data Availability

The data that support this study is available at the NASA GeneLab/NASA Open Science Data Repository with the identifiers OSD-630 (https://doi.org/10.26030/cyfk-5f38), OSD-570 (https://doi.org/10.26030/41s1-j243), OSD-572 (https://doi.org/10.26030/x57b-4722) and OSD-573 and (https://doi.org/10.26030/x57b-4722). Additional processed datasets (gene catalogs, taxonomic and gene abundances) are available at https://figshare.com/projects/Longitudinal_multi-omics_analysis_of_host_microbiome_architecture_and_immune_responses_during_short-term_spaceflight/176043. This Figshare repository additionally contains figures detailing the top most abundant taxa for each alignment algorithm before and after decontamination, and additional data, methods, and detailed crew background can be found in the collections paper, protocol papers, and additional SOMA package papers [41–51]. Select data can be visualized online through the SOMA Data Explorer: https://soma.weill.cornell.edu. The GenBank viral database used was the most recent as of 2022-07-26. The GTDB database used was the 202 release. The MetaPhlan4 database was mpa_vJan21_CHOCOPhlAnSGB_202103. The Kraken2 database contained all NCBI listed taxa (bacteria, fungal, and viral genomes) in RefSeq, as of 2022-09-01. The Phanta database was the most recent as of 2022-08-01. The Bakta databases were the most recent as of 2022-08-18. Code used to generate Figures and analyses from this project is available at the GitHub repository for code sharing and annotation: https://github.com/eliah-o/inspiration4-omics.
