# Supplementary material for: Genome and clonal hematopoiesis stability contrasts with immune, cfDNA, mitochondrial, and telomere length changes during short duration spaceflight
Source: Precis Clin Med. 2024 Apr 8;7(1):pbae007. doi: 10.1093/pcmedi/pbae007 (PMC11022651; doi:10.1093/pcmedi/pbae007)
Supplement: pbae007_Supplemental_Files [file pbae007_supplemental_files.zip › Supplemental figures.docx]

**Supplemental figures**


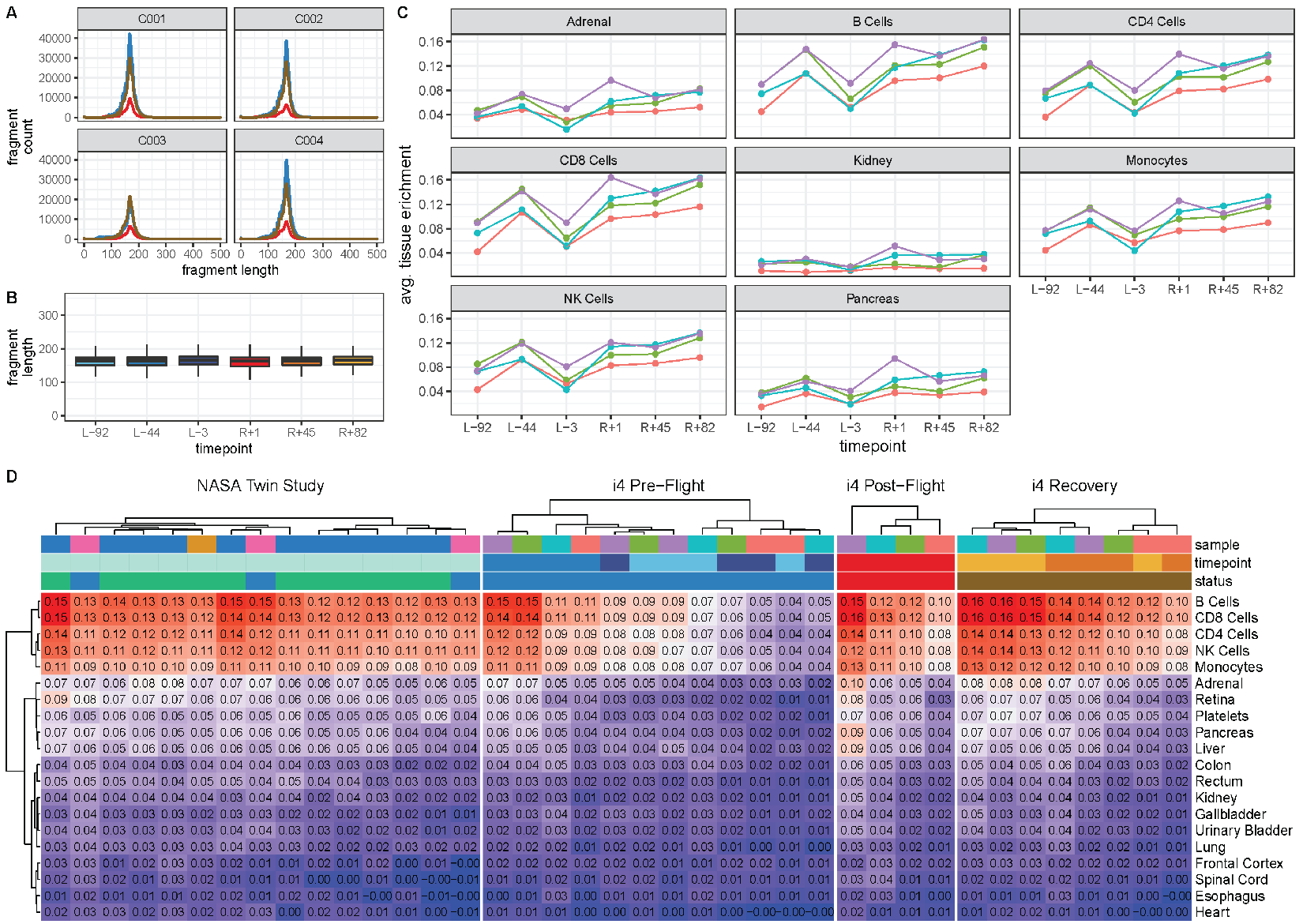


**Figure S1. Quality control of cfDNA and extended tissue-of-origin decomposition.** A&B. Size distribution of fragments shows characteristic for cfDNA peaks around 152-167bp indicating that most fragments encompass one nucleosome. The lack of enrichment of longer cfDNA fragments originating from damaged blood cells confirms the high quality of data and correct extraction of cfDNA from plasma. C&D. Extended data depicting tissue-of-origin deconvolution of cfDNA fragments based on all tissue-specific expression signatures from the Human Proteome Map highlights the similarities in I4 crew profiles (panel C, selected signatures) and a higher level of baseline heterogeneity than ground and pre-flight samples from NASA Twin Study (panel D, cite: Bezdan et al). The difference in the range of enrichment values in both studies is most likely a combination of the batch effect between studies and the change in sequencing technology. Analyzed separately we note baseline changes in expression signatures of ground-bound subjects over time in both NASA Twin Study and I4 samples.


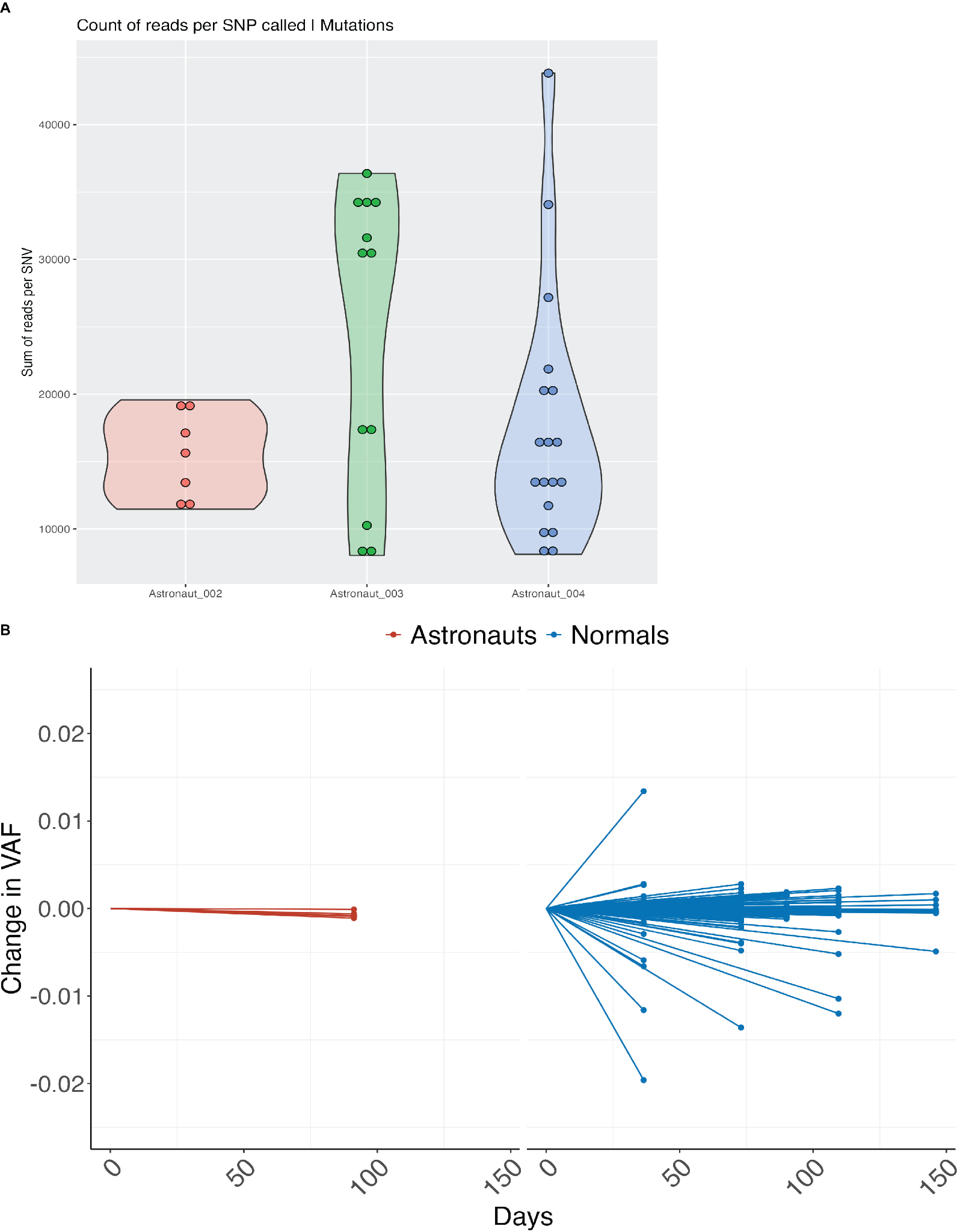


**Figure S2. Clonal Hematopoiesis.** A. Distribution of reads of all SNPs mentioned. With a mean surpassing 15,000x of coverage. B. A comparison of genomic stability of putative driver mutations for the astronauts over several months in the range of VAF +- 0-0.02 when compared to a healthy control cohort (Median age 66.5 [34.1, 88.8]. No trends or patterns of genomic instability are seen in this pre- vs post-flight comparison.


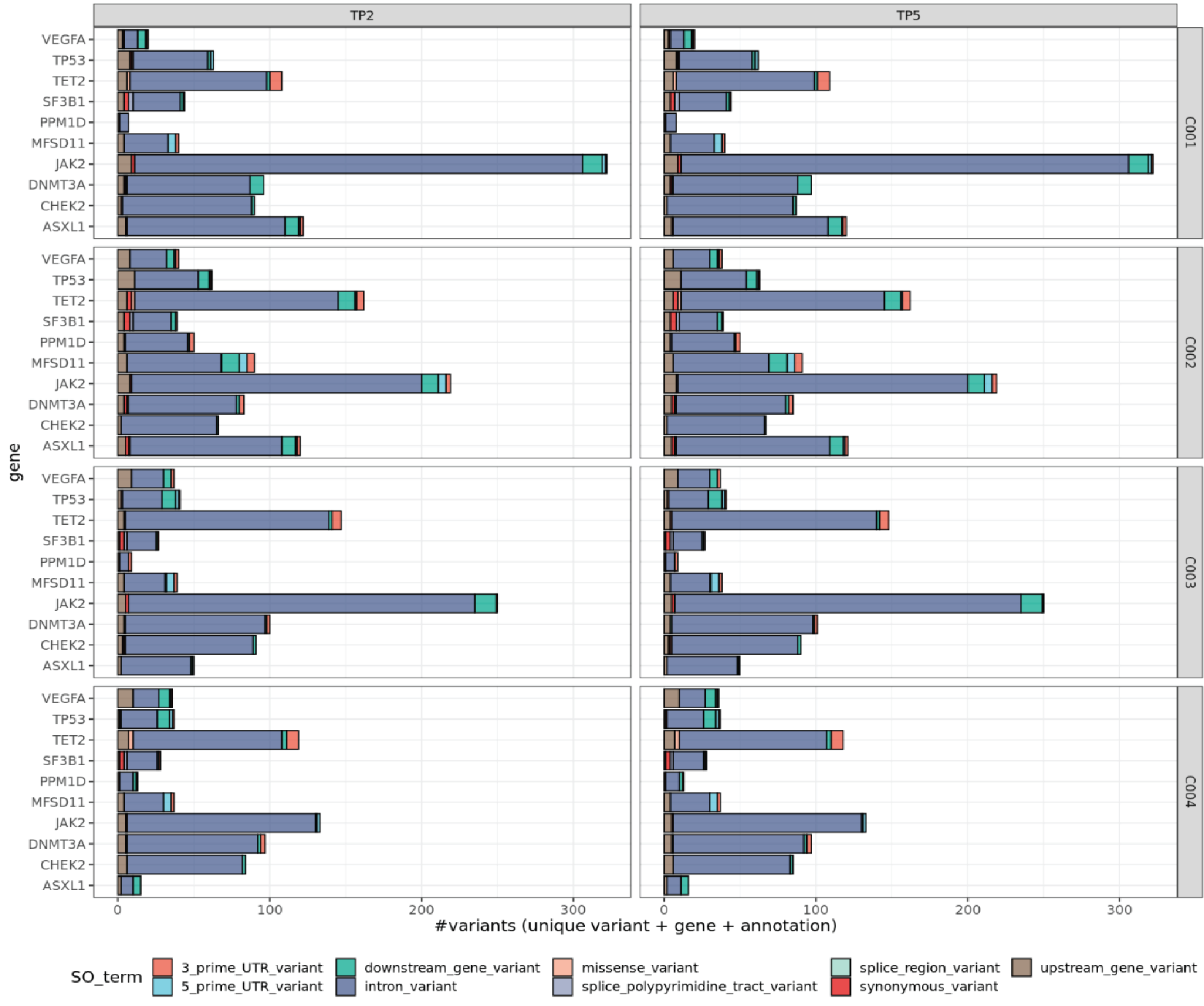


**Figure S3_A. Comparison of variant effects in WGS data supports the results for selected deep-sequencing target genes.** We report counts of unique (variant, effect annotation) pairs for selected genes and both timepoints. Denoting similar mutational profiles in pre-flight and post-flight timepoints.


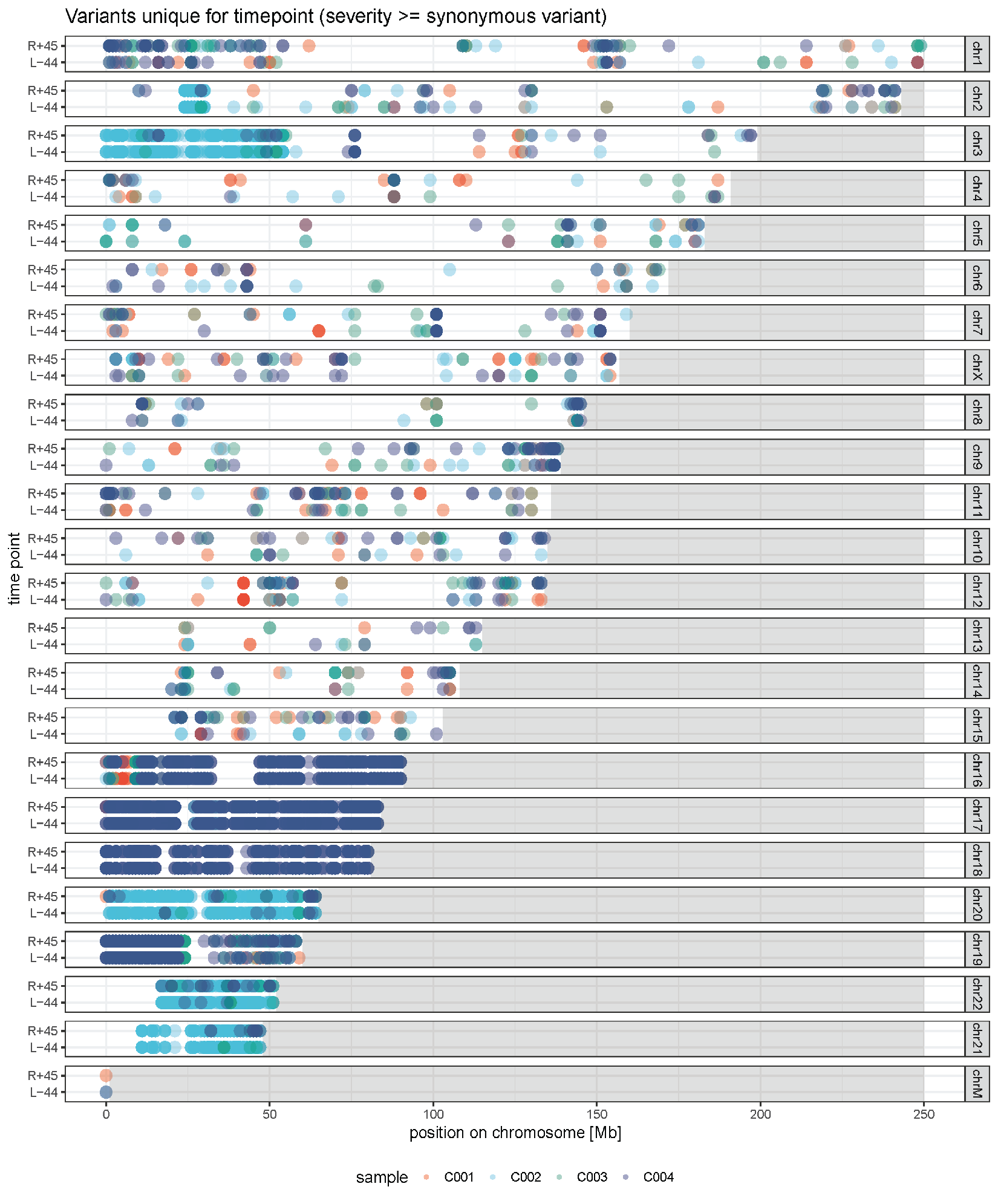


**Figure S3_B. Evaluation of variants uniquely called before and after spaceflight** Comparison of genomic coordinates of timepoint-specific variant calls reveals that the majority of variants called uniquely in R+45 have an L-44 variant counterpart in exactly the same position but with different alternative alleles. The majority of such pairs are likely to be a result of errors introduced during sequencing and do not reflect actual biological variability, supporting the results reported in Figure 3. Here, we report variants with the severity of at least a synonymous variant across the whole genome.


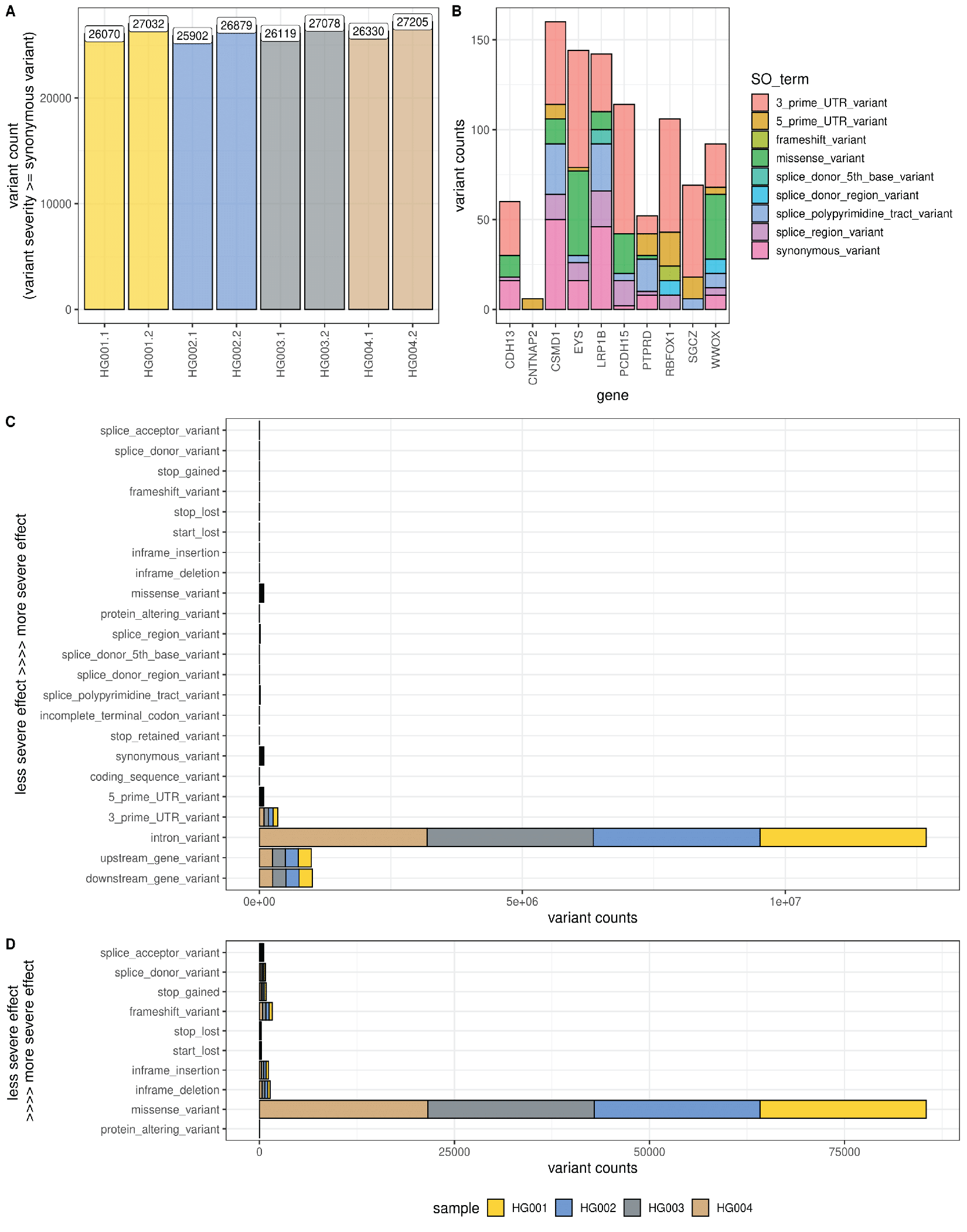


**Figure S3_C. Whole genome sequencing and variant calling pipeline evaluation using independent controls** S2A. Comparison of selected samples from Genome In a Bottle (add citation) reveals comparable mutational burdens at a genome-wide scale. We note a slight increase in the total number of variants for all samples in the second experiment (samples HG001.2, HG002.2, HG002.3, and HG004.2). Here we count variants as unique changes of reference to alternative alleles at a given position and report total counts of variants with the severity of at least a synonymous variant. S**B. The genes with the greatest mutational burden are the same for both control and i4 samples. We report unique counts of (variant, effect annotation) pairs per gene of interest. S**C. Variant effect annotation distribution of control samples mimics the one observed in i4 samples (Fig 3C&D), with the majority of mutations in non-coding regions. We report unique counts of (variant, effect annotation) pairs across the whole genome. S**D. Comparison of variant effect annotations across the whole genome for moderate to high-severity variants.


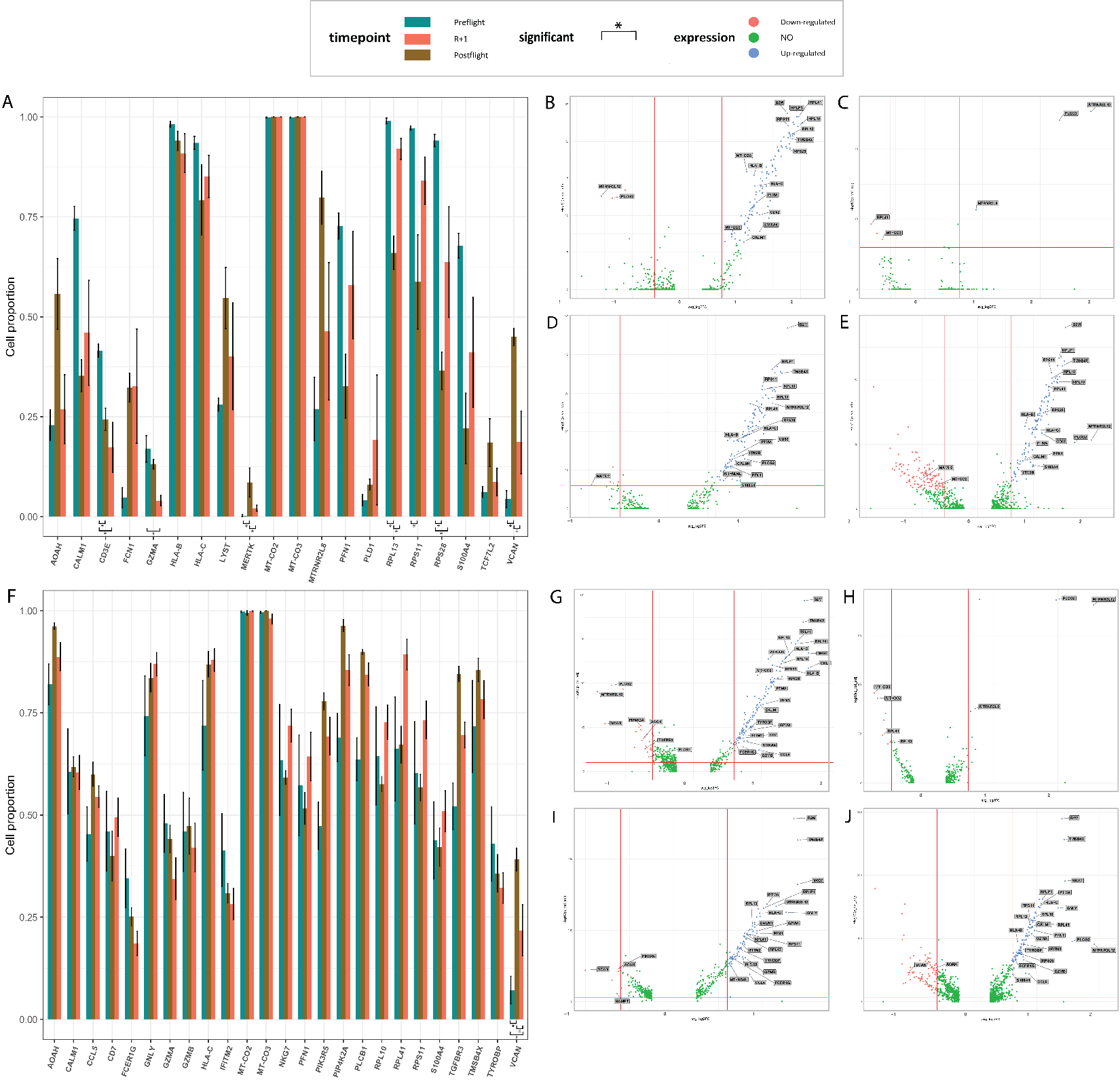


**Figure S4. Cell Proportion and Differentially Gene Expression.** 4A. Proportion of CD4 T cells expressing genes. 4B, C, D, & E. CD4 T cells volcano plot comparison of differential gene expression in L-92 vs R+1, R+1 vs R+82, L-92 vs R+82, & L-92 vs R+1 vs R+82, respectively. 4F. Proportion of natural killer (NK) cells differential expression. 4G, H, I, &, J. NK volcano plot comparison of differential gene expression in L-92 vs R+1, R+1 vs R+82, L-92 vs R+82, & L-92 vs R+1 vs R+82, respectively.

**Competing Interest Declaration**

Christopher E. Mason is a co-Founder of Cosmica Biosciences. Braden Tierney is compensated for consulting with Seed Health and Enzymetrics Biosciences on microbiome study design and holds an ownership stake in the former. Kelly Bolton receives research funding from Servier and Bristol Myers Squibb and serves on the medical advisory board of GoodCell. Kelly Blease^,^ Juan Moreno, Andrew Boddicker, Junhua Zhao, Bryan Lajoie, Andrew Altomare, Semyon Kruglyak, and Shawn Levy are employees of and have a financial interest in Element Biosciences. Irina Matei is a Co-Principal Investigator for research projects funded by Atossa Therapeutics. Caleb M. Schmidt, Julian C. Schmidt, and Michael A. Schmidt hold shares in Sovaris Holdings, LLC. Min Yu is the founder and president of CanTraCer Biosciences Inc. Authors not listed here do not have competing interests.

.
